# Crosstalk Between Hypoxia and ER Stress Response: A Key Regulator of Macrophage Polarization

**DOI:** 10.3389/fimmu.2019.02951

**Published:** 2020-01-08

**Authors:** Paula Díaz-Bulnes, María Laura Saiz, Carlos López-Larrea, Ramón M. Rodríguez

**Affiliations:** ^1^Translational Immunology Laboratory, Health Research Institute of the Principality of Asturias, Hospital Universitario Central de Asturias, Oviedo, Spain; ^2^Immunology Service, Hospital Universitario Central de Asturias, Oviedo, Spain

**Keywords:** hypoxia, UPR, macrophage polarization, immunometabolism, immune response

## Abstract

Macrophage activation and polarization are closely linked with metabolic rewiring, which is required to sustain their biological functions. These metabolic alterations allow the macrophages to adapt to the microenvironment changes associated with inflammation or tissue damage (hypoxia, nutrient imbalance, oxidative stress, etc.) and to fulfill their highly energy-demanding proinflammatory and anti-microbial functions. This response is integrated via metabolic sensors that coordinate these metabolic fluxes with their functional requirements. Here we review how the metabolic and phenotypic plasticity of macrophages are intrinsically connected with the hypoxia stress sensors and the unfolded protein response in the endoplasmic reticulum, and how these molecular pathways participate in the maladaptive polarization of macrophages in human pathology and chronic inflammation.

## Introduction

Macrophages are highly plastic immune cells involved in tissue homeostasis, immune response and inflammation. In recent years, numerous studies have demonstrated that macrophage plasticity is closely linked to a metabolic adaptation that supports the energy requirements and cellular growth associated with these functions (1). In the inflammatory milieu, stimulated macrophages face challenging metabolic conditions in which they must compete for nutrients and oxygen with other immune cells or pathogens invading the tissue. In this environment, macrophages must generate sufficient energy output to sustain growth, phagocytosis, antimicrobial functions, and also to fuel the highly demanding synthesis of inflammatory mediators that contribute to the propagation or resolution of the inflammatory response. In a simplified view, macrophage polarization states have been defined as proinflammatory M1 (classically activated) or anti-inflammatory M2 (alternatively activated), a classification that mirrors the Th1/Th2 polarization of T cells (2). The definition of these activation states contributed to the understanding and categorization of the opposing functions of macrophages: killing and repairing (3). However, in reality, the dichotomous M1/M2 distinction is not representative of what takes place in an *in vivo* setting, as M1 and M2 stimuli do not exist alone in tissues. Instead, the macrophage population represents a continuum of phenotypes that stands between these two extremes, implying that discrete populations should not be so crudely depicted (4). Indeed, transcriptome analysis of stimulated macrophages with different activation signals show that these cells experience a transcriptional reprogramming that extends the M1/M2 paradigm (5). However, since most of the preceding literature has used the nomenclature based on the M1/M2 classification as a tool for dissecting the complex macrophage phenotypes, this terminology has been maintained throughout some parts of this review.

Pathological situations in which nutrient availability is compromised, such as infection, chronic inflammation, diseases associated with metabolic/nutrient imbalance (diabetes, obesity, atherosclerosis) or ischemia/reperfusion events associated with organ transplantation or surgery, generate metabolic stress that potentially subverts macrophage functions to induce maladaptive polarization states (6–8). Macrophages can perceive these signals in the tissue microenvironment *via* metabolic sensors that coordinate metabolic and transcriptomic rewiring and are therefore very responsive to any abnormal imbalance associated with pathology. For instance, hypoxic (oxygen-limiting) environments associated with inflammation or ischemia activate cellular sensors for oxygen and the hypoxia-inducible factor (HIF), which induce a metabolic switch from oxidative to glycolytic metabolism and proinflammatory polarization that further exacerbates the inflammatory response (9, 10). This hypoxic environment is also closely linked to an endoplasmic reticulum (ER) stress response, which is critical for the integration of the metabolic and inflammatory responses in macrophages. The ER organelle plays a central role in cellular nutrient sensing, activating the signaling pathway called the unfolded protein response (UPR) under metabolic stress conditions such as hypoxia or nutrient imbalance (amino acid or glucose deprivation, infectious process, etc.). This response is partially mediated by the mTORC1 pathway, which is a positive regulator of protein synthesis, and cell growth that coordinates the cellular balance between anabolic pathways and energy consumption in macrophages (11).

Considering all this evidence, it is clear that cellular sensors for oxygen and ER stress pathways contribute critically to the signal integration and metabolic adaptation associated with various pathological conditions. In this context, macrophage polarization lies at the intersection between metabolic imbalance and inflammation, and understanding the molecular pathways connecting these processes will be critical for the development of new therapeutic strategies. Here, we review how ER stress and hypoxic responses are organized and connected with macrophage function, focusing particularly on the maladaptive polarization states associated with the pathological contexts in which the metabolic balance in macrophages is compromised.

## Molecular Mechanisms in ER Stress: Unfolded Protein Response

**Figure 1:**
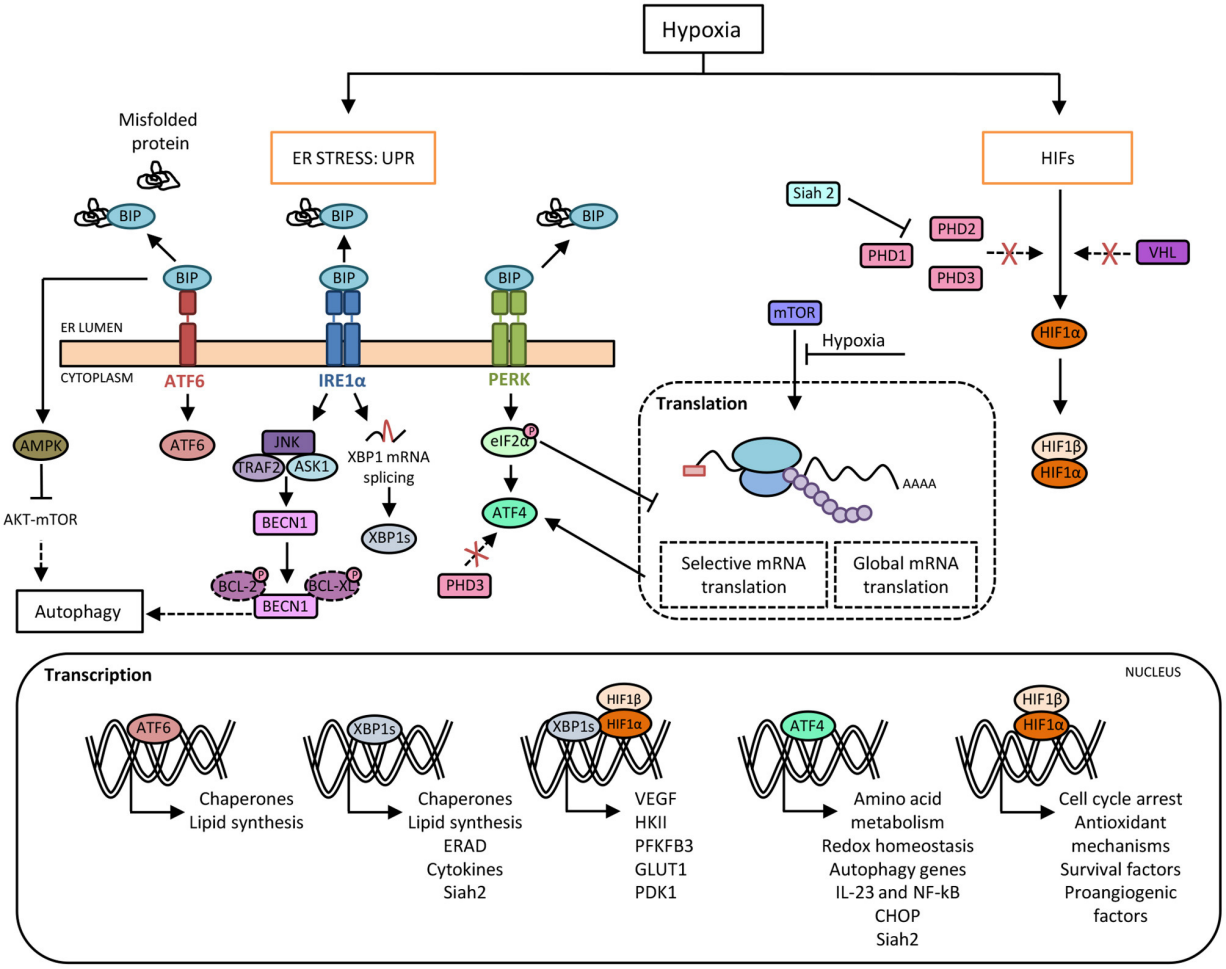
Crosstalk between hypoxia and the unfolded protein response (UPR) pathways. Hypoxia induces protein misfolding due to the lack of oxygen required for the formation of disulphide linkages, which leads to endoplasmic reticulum (ER) stress and the activation of UPR. Under basal conditions, BIP binds to PERK, IRE1α and ATF6 and prevents their activation. In response to ER stress during hypoxia, BIP dissociates from the three sensors, allowing their activation. Activated IRE1α mediates XBP1 mRNA splicing (XBP1s) that it is translocated to the nucleus and regulates the expression of genes involved in cellular maintenance pathways. Activated PERK phosphorylates eIF2α resulting in a general slowdown of protein synthesis that allows the activation of ATF4, a master transcription factor which induces the expression of target genes such as CHOP. Moreover, there is an inhibition of mTOR-dependent protein translation. Besides, HIF-1α avoids proteasome degradation promoted by prolyl-hydroxylases (PHDs) and von Hippel-Lindau (VHL) protein, which allows its nuclear translocation and the induction of genes required for adaptation to a hypoxic environment. In this context, the inhibition of PHD3 activity enables ATF4 stabilization. Furthermore, ATF4 and XBP1 upregulate the expression of the ubiquitin ligase Siah2, which in turn provokes the degradation of PHDs favoring HIF-1α expression, thus establishing another link between hypoxia and UPR pathways. Moreover, XBP1 and HIF-1α trigger a transcriptional complex that regulates the expression of genes associated with HIF-1α through the recruitment of RNA polymerase II. In addition, hypoxia-induced cell damage is counteracted by the UPR pathway through the upregulation of autophagy by ATF4 and IRE1α. Direct induction of autophagy may be also achieved by BIP-dependent activation of AMPK, which attenuates AKT-mTOR signaling.

The ER has a crucial role in maintaining cellular functions, such as protein folding, maturation and assembly of proteins that are trafficked along the secretory pathway, as well as preserving cellular calcium homeostasis. Several physiological and pathological conditions involving imbalance in ER folding capacity, accumulation of misfolded proteins, hypoxia, amino acid or glucose deprivation, oxidative stress, viral infection or disruption of ER calcium balance can trigger ER stress and activate the UPR that maintains cellular homeostasis and cell survival (12). This mechanism rescues the cells from the damage caused by ER stress, and in the event of unresolvable stress, induces apoptosis. The UPR comprises three major signaling pathways, which are initiated by the activation of three protein sensors: activating transcription factor 6 (ATF6), pancreatic eukaryotic translation initiation factor 2α (eIF2α) kinase (PERK), and inositol-requiring enzyme 1α (IRE1α). Under normal conditions, these sensors are bound to glucose-regulated protein 78 (GRP78), an ER chaperone, also known as BiP (binding immunoglobulin protein), that maintains them in an inactive state. Under ER stress conditions, GRP78 dissociates from the sensors and binds to unfolded proteins (13), allowing activation by dimerization or translocation (Figure 1). Accordingly, activated IRE1α performs two enzymatic functions upon dimerization: serine/threonine kinase and endoribonuclease (RNase) activity (14). This RNase domain in IRE1α initiates the non-conventional splicing of XBP1, which produces a translational frameshift and creates XBP1s (spliced), a potent transcriptional activator (15). XBP1s is translocated to the nucleus and induces the transcription of an extensive variety of chaperones and enzymes that together increase ER size and function. During prolonged ER stress, the beneficial effects of IRE1α activation through the activity of XBP1s may be reduced and could activate the properties of IRE1α to promote inflammation and cell death (16). Similar to IRE1α, dissociation of GRP78 from the ER luminal domain of PERK induces activation through homodimerization and autophosphorylation. Activated PERK phosphorylates the eukaryotic translation initiation factor (eIF2α), which reduces the degree of recognition and translation of the AUG initiation codon, resulting in a lower protein load on the damaged ER (17). Remarkably, eIF2α can be phosphorylated independently of ER stress by the general control non-derepressible 2 (GCN2) kinase, which is induced by amino acid deprivation (18). This results in the activation of ATF4, Nrf2 (nuclear erythroid 2 p45-related factor 2), and NF-κB (nuclear factor kappa β), a master transcription factor with numerous functions, including regulation of the inflammatory response (19). ATF4 also activates the transcription of C/EBP homologous protein (CHOP), which is involved in ER-stress-mediated apoptosis and cell death (20). The third arm of the pathway relies on ATF6, a type II transmembrane protein. There are two isoforms of ATF6 (ATF6α and ATF6β), but only ATF6α is required to activate UPR. When ATF6 senses ER stress, it is transported to the Golgi, where it is cleaved by site 1 and site 2 proteases (S1P and S2P) (21). The cleaved soluble cytosolic fragment can now enter the nucleus and trigger the expression of genes encoding proteins such as GRP78, GRP94, p58IPK, and XBP1 that function to increase ER capacity and folding as well as the expression of genes of the ERAD pathway involved in protein degradation (22, 23). Of note, cleaved ATF6 can act as an enhancer to increase the CREBH-mediated (cAMP response element-binding protein H) acute inflammatory response (24), representing a link between ATF6 and inflammation.

## UPR in Macrophage Physiology and Function

It is generally accepted that ER stress and inflammation are two mechanisms that allow tissue and cells to resolve infections or stress-induced situations. Various studies have demonstrated the crosstalk between these pathways and show that the UPR route is essential to ensure the normal physiology of dendritic cells and macrophages. Toll-like receptor (TLR) signaling induces ER stress in macrophages, which in turn amplifies the responses after the binding of the TLRs to their ligands (25). TLR2 and TLR4 induce the activation of IRE1α, and consequently of XBP1s, through a mechanism that needs the NADPH oxidase NOX2 and TNF receptor-associated 6 (TRAF6). The knockdown of IRE1α in J774 macrophages cells, prevents the production of IL-6, TNF-α, and interferon β (IFN β). This result shows the crucial effect of the IRE1α-XBP1s axis on the TLR-mediated response (26). Moreover, several studies have reported that ER stress activates glycogen synthase kinase (GSK)-3β, accelerating inflammation and lipid accumulation (27, 28). Experiments performed on RAW264 macrophages revealed that the IRE1α pathway is involved in the increase in IL-1β production through GSK-3β activation. In addition, pharmacological inhibition of GSK-3β shows that GSK-3β inhibits XBP1 splicing, entailing a decrease in TNF-α expression and possibly in TLR signaling (29).

On the other hand, it is known that ATF4 directly binds the IL-6 promoter linking the PERK branch with inflammation (30). The involvement of this UPR branch in inflammation has also been noted in another study in human macrophages, in which the induction of the ATF4-CHOP branch was attenuated by TLR signaling in a TRIF (TIR-domain containing adaptor inducing interferon-β) dependent way (31), whereas the other UPR pathways were not suppressed. Interestingly, TLR engagement did not impede phosphorylation of PERK or eIF-2α, but phospho-eIF-2α failed to promote translation of the CHOP activator ATF4 because TLR-TRIF signaling enhances eIF2B-GEF activity to counteract the effects of p-eIF2α (32). In this way, the beneficial aspects of prolonged physiological ER stress can be achieved, avoiding the negative effects of prolonged CHOP expression because deficiencies in this pathway allow the macrophages to survive, potentiating the immune response. The ATF6 branch synergizes with TLR stimulation in liver macrophages, enhancing NF-κB signaling in the context of liver ischemia reperfusion (33). ER stress in murine bone marrow-derived macrophages (BMMs) induced by chemicals and followed by TLR stimulation yields significantly higher levels of TNF-α and IL-6, but lower levels of IL-10. Moreover, ATF6 KO liver macrophages showed that TNF-α and IL-6 depend on ATF6 in response to TLR4 activation. In addition, the AKT anti-inflammatory signaling pathway is inhibited by the ER stress response in BMMs, and the absence of ATF6 is enough to restore its activation and enhance NF-κB signaling (33).

Finally, in an experimental model of lung injury and fibrosis, GRP78 heterozygous mice have fewer lung macrophages due to their higher caspase-3 and CHOP levels compared with those of wild type (WT) mice. It should be noted that an increase in the frequency of T cells induced by bleomycin was associated with an increment in M2 macrophages in this lung damage mouse model (34). CHOP deficiency in mice allows macrophages to accumulate by inhibiting ER stress-mediated cell death, as assessed in experimental models of liver and pulmonary fibrosis, suggesting that GRP78 protects from lung fibrosis while up-regulation of CHOP promotes it (35). The IRE1-XBP1 arm of the UPR is involved in the synergy of IL-6 with IL-4 and IL-13 in profibrotic “M2-like” hyperpolarization in human and mouse cells. On the basis of all these findings, the authors proposed that an inhibitor of the IRE1-XBP1 axis might be beneficial in preventing the profibrotic capacity of macrophages (36).

**Figure 2:**
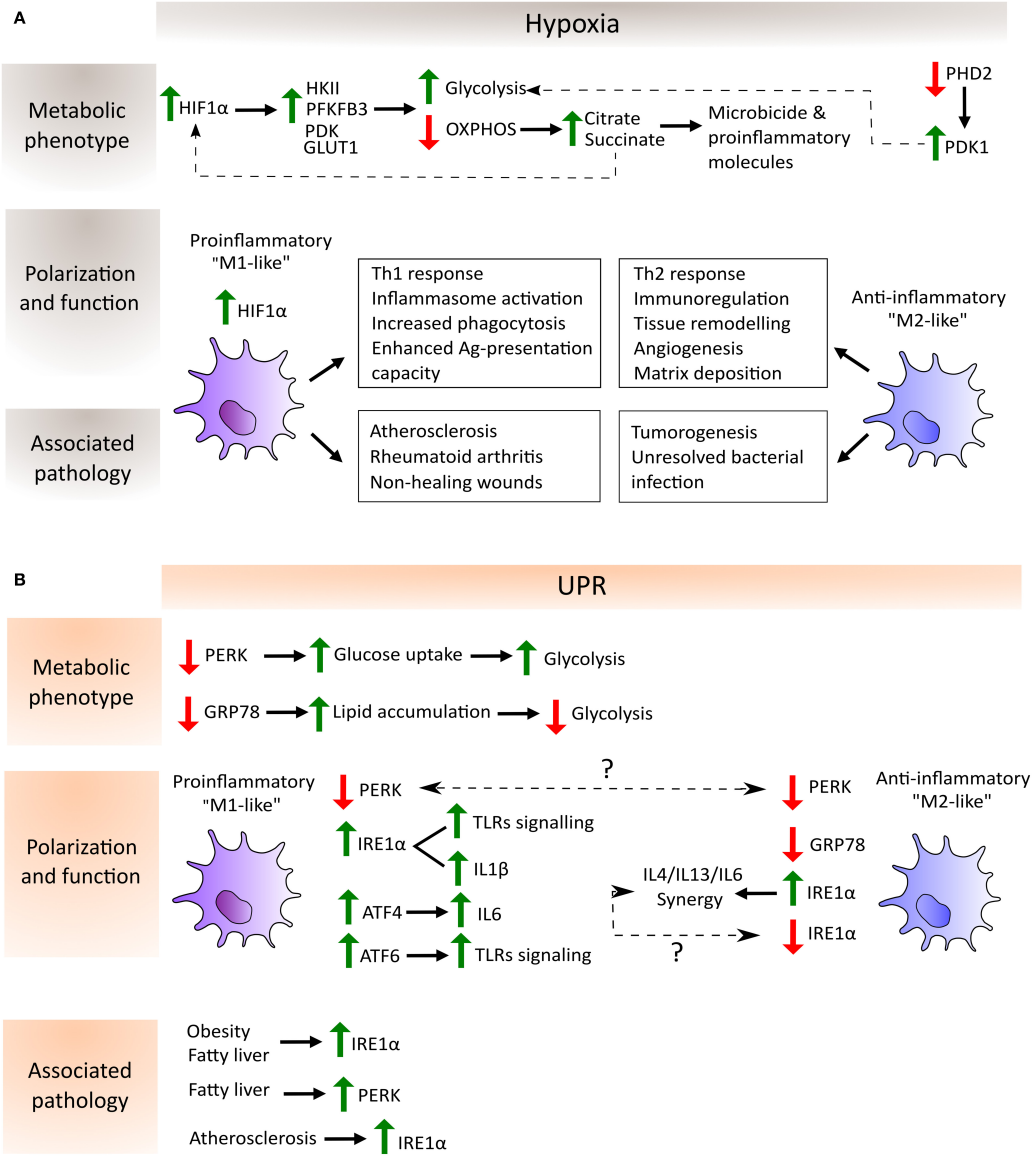
Schematic depiction of the regulation of macrophage metabolic phenotype, polarization and function by hypoxia **(A)** and UPR-dependent **(B)** pathways as well as their association with the corresponding pathological scenario. **(A)** In hypoxia, HIF-1α avoids degradation and translocate to the nucleus to induce the expression of glycolytic enzymes such as hexokinase (HKII), phosphofructokinase (PFKFB3) and pyruvate dehydrogenase kinase (PDK), and glucose transporters such as GLUT1. On the other hand, the inactivity of prolyl-hydroxylase 2 (PHD2) during hypoxia entails the increase of PDK1 which further contributes to the glycolytic metabolic switch. Moreover, OXPHOS shutdown provokes the accumulation of the TCA cycle intermediates citrate and succinate which are employed by the macrophage to produce microbicide and proinflammatory molecules. Succinate in turn increases the activity of HIF-1α. These metabolic adaptations potentiate proinflammatory macrophage polarization, characterized by their increased capacity to mount Th1 responses, increased phagocytic and antigen presentation capacities and enhanced inflammasome activation. In contrast, anti-inflammatory macrophages are involved in processes related to the resolution of the inflammatory response and homeostasis reestablishment. Lastly, hypoxia can be found in several pathological situations, where the balance between pro- and anti-inflammatory macrophages determines disease outcome. Proinflammatory macrophages are key mediators in atherosclerosis and autoimmune diseases such as rheumatoid arthritis. Their accumulation in non-healing wounds has been associated with the chronification of inflammation in the wound. However, these macrophages are required to mount proper anti-tumor and anti-bacterial responses, where a shift toward an anti-inflammatory phenotype has been shown to be detrimental. **(B)** Increased lipid accumulation and ATGL activity promotes “M2-like” macrophage polarization in the absence of GRP78 (also known as BIP), whereas decreased ATGL activity and elevated glucose uptake, leading to enhanced glycolysis to favor “M1-like” macrophage polarization, have been detected in the absence of PERK. Conversely, PERK knockdown induces an “M2-like” polarization state, although the explanation of this discrepancy requires further experimentation (indicated by a question mark in the picture). The same happens with respect to the IRE1α arm of the UPR where, on one hand, its activation is necessary for IL6 synergy with IL4 and IL13 to enhance anti-inflammatory macrophage polarization and, on the other hand, its increased activation in adipose tissue macrophages (ATMs) and in bone marrow-derived macrophages (BMMs) leads to a shift to a proinflammatory state. Typical features of proinflammatory macrophages, such as TLR signaling, inflammasome activation and proinflammatory cytokines expression, are enhanced by the activation of the three arms of the UPR: IRE1α and ATF6 synergize with TLR signaling, IRE1α is involved in the increase in IL1β through GSK3β activation, and ATF4 directly binds to the IL6 promoter. Finally, metabolic disorders such as obesity, fatty liver and atherosclerosis induce an increase of the IRE1α and PERK pathways of the UPR in macrophages, highlighting the link between a metabolic imbalance and maladaptive macrophage polarization states.

The influence of UPR inhibition was analyzed in RAW 264.7 macrophages in which specific knockdown was performed in each UPR signaling arm individually (GRP78, PERK, and IRE1α). These experiments illustrated the differential effects on macrophage polarity due to differential activity in an adipose triglyceride lipase (ATGL), which cleaves triacylglycerol to generate non-esterified fatty acids. GRP78 knockdown in macrophages stimulated with LPS exhibits elevated ATGL levels relative to PERK knockdown macrophages. The same study demonstrated that PERK inhibition prompts an elevated glucose uptake in macrophages independently of LPS stimulation. Metabolic flux analysis demonstrated that IRE1α and PERK inhibition increases mitochondrial metabolism and basal glycolysis in contrast to GRP78 inhibition, which reduces the values of these two parameters (37). All these studies demonstrate the importance of UPR pathways in innate immune functions (Figure 2).

## Molecular Mechanisms in Hypoxia

Aerobic organisms need oxygen for mitochondrial respiration and a wide range of other metabolic processes, for which reason sophisticated mechanisms for sensing changes in oxygen tension have evolved to modulate wide-ranging aspects of cell behavior. Hypoxia is a condition in which the oxygen supply is reduced relative to demand, causing oxygen levels to drop below 1%. Although it is mainly associated with pathological situations such as cancer, cardiovascular disease, dementia and diabetes, it can also be part of normal physiology, for example, during fetal development (38). During hypoxia, cells experience a reduction in mitochondrial ATP production due to lack of oxygen, which is the terminal electron acceptor in the mitochondrial electron transport chain (ETC) required to generate energy by oxidative phosphorylation (OXPHOS). Under these circumstances, cells switch their metabolism to glycolysis to obtain energy in an oxygen-independent manner, although this is at the cost of a poorer ATP yield (39). In addition, hypoxic cells are threatened by the accumulation of excessive reactive oxygen species (ROS) arising from the mitochondria (40). Thus, to survive at low levels of oxygen, cells must reduce energy consumption, temporarily arrest the cell cycle, upregulate antioxidant mechanisms to decrease ROS excess, and secrete survival and proangiogenic factors (41–44). Cells manage to develop all these adaptations in ways that are essentially mediated by hypoxia-inducible factors (HIFs) (41) and by inducing pathways that result in the general slowdown of processes involving oxygen and energy consumption, which mainly involve the UPR (45) and mTOR (46) pathways. These pathways synergize to activate common downstream targets and may also influence one another, such as in the case of mTOR and HIF-1α mutual regulation (47).

### Transcriptional Response to Hypoxia: HIFs

Hypoxia-inducible factors, HIFs (HIF1, 2, and 3) are dimeric transcription factors, which consist of an α subunit with oxygen-dependent instability and a more stable, constitutively expressed β subunit. The family members HIF-1 and HIF-2 are the main hypoxia sensors (48). The exact role of HIF-3 requires further investigation although it is thought to induce a unique transcriptional program in response to hypoxia as well as acting as a dominant-negative regulator of HIF activity (49). HIF-1α is ubiquitously expressed in all cells, whereas HIF-2α and HIF-3α are specifically expressed in certain tissues, particularly those that are highly vascularized (50). Under conditions of normal oxygen availability, HIF-α isoforms are constantly expressed and degraded in a process that involves hydroxylation at two proline residues by specific prolyl-hydroxylase domain-containing proteins (PHDs). These enzymes act as “oxygen sensors” in the cell, as oxygen is an obligatory substrate for their catalytic activity. Following hydroxylation, HIF-α binds to the von Hippel–Lindau (VHL)-E3-ubiquitin ligase complex for ubiquitination and rapid degradation by proteasome (51). A second oxygen sensor that regulates HIF-α function is the asparaginyl hydroxylase FIH (factor-inhibiting HIF) enzyme, which blocks the interaction of HIF-α with its transcriptional coactivators p300/CBP, thereby establishing another layer of regulation and impairing residual HIF transcriptional activity (52, 53). Therefore, under hypoxia, HIF proteins avoid degradation and translocate to the nucleus where the active heterodimers bind to hypoxia-response elements (HREs) on the promoter or enhancer regions of target genes, causing their transcriptional activation. Examples of these target genes include some of those involved in angiogenesis, cell proliferation/survival, glucose and iron metabolism (54, 55) (Figure 1).

### Hypoxia-Induced Inhibition of Protein Synthesis: UPR and mTOR

During hypoxia, apart from the HIFs-mediated transcriptional responses, there is a great reduction in the rates of global protein synthesis (they have been calculated to drop to about 7% of that of normoxic cells) that presumably reduces energy demands when oxygen and ATP levels are low. Since protein synthesis is one of the most energy-consuming cellular processes, protein translation arrest is crucial for cellular adaptation to a hypoxic environment (56, 57). Hypoxia induces protein synthesis shut-off primarily at the level of the initiation step, which allows the rapid shutdown of translation. This control takes place principally at two stages, the phosphorylation of the α subunit of eIF2 (eukaryotic initiation factor 2) and the disruption of the eIF4F complex, whose availability depends on the phosphorylation of the inhibitory protein 4E-BP1 (eukaryotic initiation factor 4E-binding protein 1) (58). The contribution of each of them largely depends on the extent (time and oxygen levels) of hypoxia. Interestingly, this differential regulation allows for a specific control of gene expression. In this way, it has been suggested that preferential mRNA translation during acute hypoxia is less reliant on eIF2α availability, whereas during prolonged hypoxia gene transcription is less dependent on eIF4F (59).

### UPR: Phosphorylation of eIF2α

One important translation regulation pathway is initiated in the endoplasmic reticulum (ER). Intracellular depletion of oxygen can result in the accumulation of misfolded proteins in the ER, as oxygen works as the major electron acceptor at the end of the formation of disulphide linkages (60), so the accumulation of unfolded proteins within the ER provokes the induction of the UPR. In fact, hypoxic-derived ROS specifically activates this pathway (61). Under hypoxia, the ER-resident kinase PERK becomes hyperphosphorylated, and in turn phosphorylates eIF2α on ser51, resulting in a general slowdown or inhibition of new protein synthesis (45). This response is part of a common adaptive pathway to restore cellular homeostasis called the integrated stress response (ISR), which relies on eIF2α phosphorylation as a means to stop protein translation abruptly upon different environmental stressors (62).

### mTOR Inhibition: eIF4F Complex Disruption

The other major pathway controlling protein translation under hypoxia initiates in the cytoplasm and involves the inhibition of the kinase mTOR. This kinase is central to the control of translation in response to stress and nutrient deprivation (63). Hypoxia triggers the inhibition of mTOR and subsequent hypophosphorylation of its substrates 4E-BP1 and S6K, promoting the inhibition of protein translation through eIF4F. This hypoxia-induced inhibition of mTOR prevails over growth factors and nutrients (mTOR activating signals) in a HIF-1α-independent manner (46). However, a mutual regulation of mTOR and HIF-1α has been noted. For instance, HIF-1α interacts with Raptor (regulatory-associated protein of mTOR) through a mTOR signaling (TOS) motif located in the N-terminus of HIF-1α, and this interaction is required for it to bind to the co-activator CBP/p300 (64). On the other hand, dampening mTOR signaling during hypoxia can be induced by HIF-1α-dependent mechanisms. For instance, the induction of REDD1, a HIF-1α target gene, inhibits mTOR signaling during hypoxic stress by acting upstream the mTOR inhibitory TSC1/TSC2 complex (65). Moreover, hypoxia results in Ataxia Telangiectasia Mutated (ATM)-dependent phosphorylation of HIF-1α on serine 696, which is required to ensure its stability, which leads to increased REDD1 transcriptional levels and again, to suppression of mTORC1 signaling (66). The relevance of this crosstalk between mTOR and HIF-1α in macrophages is exemplified by its requirement for the metabolic switch toward aerobic glycolysis as the metabolic basis of trained immunity (10).

## The Impact of Hypoxia on Macrophage Phenotype-Shaping

Macrophages are confronted by differential oxygenation levels under several pathological conditions. Hypoxic areas can be found in inflamed, infected and ischemic tissues, as well as in regions where local vessel growth cannot maintain an adequate blood supply due to cellular overgrowth, as occurs in adipose and malignant tissues (67). In these varied settings, macrophages must adjust their metabolic requirements to generate energy in an oxygen-independent fashion. Numerous studies have demonstrated that proinflammatory macrophages are highly dependent on glycolysis and that anti-inflammatory and pro-homeostatic macrophages show a stronger preference for mitochondrial OXPHOS to generate ATP (68). Hypoxia is a master driver of glycolysis, since oxygen deficit results in limited OXPHOS and cells rely on glycolysis to generate ATP. HIF-1α is fundamental to this process, which induces the expression of glycolytic enzymes such as hexokinase II (HKII) (69), phosphofructokinase (PFKFB3) (70) and glucose transporters such as GLUT1 (71). HIFs can also upregulate the expression of the enzyme pyruvate dehydrogenase kinase (PDK), which in turn provokes a drop in the conversion rate of pyruvate to acetyl CoA by the action of the enzyme pyruvate dehydrogenase (PDH), which prevents the entry of glucose into the TCA cycle (72). In this way, hypoxia-mediated activation of HIFs increases the glucose/carbon flux to the glycolytic route, but minimizes the flux through the TCA cycle and OXPHOS. Other oxygen sensors, such as prolyl-hydroxylases (PHDs) upstream of HIF, also directly contribute to the glycolytic reprogramming of macrophages. To take a specific example, the PHD2 isoform has been found to be important for regulating the glycolytic switch by altering PDK1 activity, as demonstrated by specific PHD2 deletion in the macrophage lineage *in vivo* (73). The shutdown of the TCA cycle under hypoxic conditions due to the lack of oxygen and to the effects of NO on the mitochondrial ETC causes intermediates, such as citrate and succinate, to accumulate. Citrate is then exported from the mitochondria into the cytosol, where it is converted to acetyl-CoA for use in several biosynthetic pathways and in the formation of microbicide and proinflammatory molecules. In the case of the metabolite succinate, this can be further oxidized to generate ROS and to increase the activity of HIF-1α (74–77). All these metabolic adaptations allow proinflammatory macrophages to develop their functions in the inflamed hostile microenvironment (Figure 2). Glycolysis can also take place in the presence of oxygen, a process known as the Warburg effect. This phenomenon was first detected in tumor cells, but was soon also observed in macrophages, since glycolysis serves not only as an adaptation to the hypoxic microenvironment, but also as a rapid means of obtaining energy, macromolecules for the catabolic cellular pathways, and of reducing equivalents such as ROS and NO, which are required for bactericidal activity (78).

Remarkably, HIF-1α activation can occur under normoxia, which allows the initiation of an inflammatory response before the tissue becomes hypoxic. Stimulation of macrophages with LPS increased HIF-1α protein levels and the formation of the functional HIF-1 complex (79). The induction of HIF-1α in the context of inflammation was soon found to depend on the presence of NF-κB (80). A relationship between HIF-1α glycolytic reprogramming and macrophage migratory activity through the HIF-1α/PDK1 axis was also discovered (81). HIF-1α is also recruited to the CXCR4 promoter, which mediates the chemotactic responses to its ligand CXCL12, which is expressed in hypoxic environments (82). Conversely, M2 macrophages do not need this rapid switch to glycolysis and obtain much of their energy from fatty acid oxidation and oxidative metabolism, which can be sustained for longer periods. This is consistent with their functional roles, as they appear later in the inflammatory response during the resolution phase and fulfill longer-term functions such as angiogenesis and extracellular matrix remodeling. HIF-1α and HIF-2α isoforms are differentially expressed in pro- and anti-inflammatory macrophages, respectively, according to their induction by Th1 and Th2 cytokines and by their regulation of inducible NOS2 (“M1-like”) and arginase 1 (“M2-like”) (83). Collectively, these findings demonstrate that metabolic adaptation is central to the polarization and functional activity of macrophages during hypoxia (Figure 2).

In conjunction with these metabolic changes, hypoxia is also involved in several other macrophage functions such as inflammasome activation, phagocytosis, antigen presentation and trained immunity. In hypoxic microenvironments IL-1β activity is enhanced through an increase in the synthesis of pro-IL1β and its processing enzyme, caspase-1, in LPS-stimulated human macrophages (84). Moreover, hypoxia boosts phagocytosis in macrophages via the activation of a p38 MAP kinase-HIF-1α link (85) and IFNγ-dependent upregulated expression of phagocytic receptors (86). Related with this process, hypoxic macrophages display an enhanced antigen-presentation capacity through the upregulation of costimulatory and antigen-presenting receptor expression (86, 87) (Figure 2). Innate immune cells such as macrophages have recently been found to be able to mount a memory response by a process known as trained immunity. This mechanism relies on major epigenetic changes upon initial stimulation that make the cells more responsive to subsequent stimuli (88). Induction of trained immunity is accompanied by significant changes in cellular metabolism. Indeed, the accumulation of several TCA metabolites, such as succinate and fumarate, could modulate the epigenetic landscape required for priming (89, 90). Elevated glycolysis is a central feature of this phenomenon, which, in turn, is dependent on the activation of mTOR through the dectin-1–Akt–mTOR–HIF-1α pathway (10). Oxygen may also directly regulate epigenetic enzymes such as histone demethylases (91), which are important in macrophage physiology (92). However, the exact link between these enzymes and oxygen deprivation in the context of trained immunity or macrophage polarization has not yet been assessed. On the other hand, TCA-intermediate metabolites could in turn influence HIF-1α stabilization in macrophages through their influence on the activity of hydroxylases such as succinate, which inhibits PHDs (77). It is of particular note that glycolysis-derived metabolites like lactate are known HIF-1α stabilizers (93). Specifically, lactate is an important mediator of VEGF expression in macrophages (94), where it induces Arg1 and other anti-inflammatory genes in tumor associated macrophages (TAMs) in an HIF-1α dependent way. This contributes to the development of a pro-homeostatic programme from which cancer cells may benefit to promote their growth (95).

## Metabolic and Signaling Crosstalk Between UPR and Hypoxia

It is important to note that although the aforementioned oxygen-sensitive signaling pathways are activated independently, there is growing evidence that they may also act in an integrated way, influencing each other and common downstream targets that affect gene expression, metabolism and cell survival.

### Activation of the UPR Machinery

As mentioned above, global dampening of protein synthesis takes place during hypoxia. The increase in the phosphorylation of eIF2α is a means by which the ISR contributes to this shutdown, but, at the same time, this increase activates ATF4, which plays an important role by transcriptionally regulating antioxidant genes and several others such us CHOP and GADD34, which induce apoptosis and recovery from the ER stress response, respectively (96). Mechanisms induced during the hypoxic response, such as the inhibition of PHDs, also regulate the function of the UPR protein ATF4. Specifically, the HIF hydroxylase PHD3 interacts with ATF4 and, upon hypoxia or PHD3 inhibition, is made more stable, thus establishing a link between the two adaptive systems (97) (Figure 1). On the other hand, hypoxia could activate the other two arms of the UPR pathway mediated by ATF6 and IRE1, although the evidence for this is not as robust as that for the PERK arm of the UPR. ATF6 induces cyclophilin B in gastric cancer cells in response to hypoxia, a protein which plays important roles in the attenuation of apoptosis in response to ER stress-mediated cell death (98), and loss of XBP1 inhibits tumor growth due to the reduced ability to survive in a hypoxic environment (99).

### Autophagy

An important mechanism by which UPR bypasses hypoxia and oxidative stress damage consists of the induction of autophagy. Autophagy is a conserved intracellular pathway in which dysfunctional cellular organelles are “digested” by endogenous enzymes to recycle and reuse their components. Basal levels of autophagy exist in every cell, but under stress conditions like nutrient deprivation or hypoxia, its rate increases as a cytoprotective measure (100). The mechanisms by which the UPR induces autophagy require further investigation, although ATF4 and CHOP are known to transcriptionally regulate a large array of autophagy genes according to the stress intensity (101). In addition, UPR pathways can activate AMPK, which attenuates AKT–mTOR signaling to enhance autophagy (102). Other studies showed that abrogation of UPR-mediated signaling resulted in loss of autophagic capacity and sensitized cells to hypoxia-induced cell death (103–105). Hypoxia increased transcription of the essential autophagy genes MAP1LC3B and ATG5, through the transcription factors ATF4 and CHOP, respectively (Figure 1). This observation was found in two distinct tumoral cell lines, colorectal adenocarcinoma and glioblastoma-astrocytoma, indicating that this mechanism is a feature shared by tumorgenicity processes (103). Moreover, the finding that pro-survival autophagy could be induced by the UPR under hypoxic conditions, was also noted in a non-tumoral context. In H9C2 cells (cardiomyocyte cell line), the activation of autophagy secondary to the stimulation of the UPR alleviated cell apoptosis during hypoxia/reoxygenation injury (105). In alveolar macrophages, rapamycin induced autophagy, results in decreased macrophage apoptosis by reducing ER and oxidative stress in the early stages of hypoxia (106), highlighting the complex relationship between the UPR route and autophagy. Finally, autophagy in macrophages is known to reshape metabolism in concert with HIF-1α and mTOR pathways (107, 108). However, it is not yet known whether targeting individual UPR components can impair the necessary metabolic rewiring of macrophages through autophagy impairment under hypoxic conditions.

### Other Molecular Interactions

Given that hypoxia induces the UPR, and that this adaptation to hypoxic environments relies on dramatic changes occurring in cellular energy metabolism, it is important to explore the role of the UPR pathways in modulating macrophage metabolism. ATF4, a downstream component of the PERK signaling pathway, has recently been implicated in the HIF1-dependent induction of carbonic anhydrase 9 (CA9) during hypoxia (109), which is required to resist the low pHs that occur in the hypoxic compartments of tumors (110). The heterodimerization of HIF-1α and XBP1s has been described in breast cancer cell lines, exemplifying the cooperation between UPR and hypoxia. ChIP-seq experiments revealed statistically significant enrichment of both HIF-1α and XBP1 motifs, suggesting frequent colocalization of HIF-1α and XBP1 to the same regulatory elements (Figure 1). Though not related to metabolic functions, this coregulation of the two transcription factors is involved in the maintenance of stem cancer cell activity (111). For this reason, it would be interesting to explore whether this interaction also occurs in immune cells during hypoxia or if it is only a malignant feature. Some genes expressed during hypoxia, such as VEGF, can be activated independently of HIF-1α by the three arms of the UPR (112). Another protein, the ubiquitin ligase Siah2, is another example of the cooperation of HIF and UPR responses during hypoxia. Siah2 promotes the degradation of PHDs through the action of the proteasome, stabilizing HIF-1α in hypoxia (113). In turn, this ubiquitin ligase can be transcriptionally upregulated by ATF4 or XBP1s under ischemia (114) (Figure 1). However, this cooperative mechanism in macrophage polarization remains to be explored.

## Involvement of the UPR and Hypoxia Pathways in the Maladaptive Polarization of Macrophages in Pathology and Chronic Inflammation

### ER Stress, Macrophages Polarization, and Metabolic Disorders

Metabolic disorders have increasingly been recognized as a health threat leading to chronic diseases such as type 2 diabetes, atherosclerosis and obesity. These diseases have different physiological symptoms but all of them share some traits, such as intracellular stress and inflammation. In this inflammatory context, macrophage plasticity and metabolism can be modulated by pathological microenvironments, such as those of metabolic syndromes and cancer. Extrinsic factors in these tissues derived from an altered metabolism directly influence the metabolic adaptation of macrophages. Indeed, the link between metabolic shaping of macrophages and their functional plasticity has been highlighted by several transcriptomic and metabolomic studies (74). For instance, ROS, cytokines, hormones, lipids, metabolite derivatives such as lactate or fatty acids derived in the metabolically altered tissues directly influence macrophage rewiring. This setting fosters the activation of the UPR pathway, which further contributes to disease progression (Figure 2).

#### Obesity

The primary function of adipose tissue is to store excess nutrients as triacylglycerols and to release free fatty acids during fasting. This tissue secretes adipokines, which have a role in several physiological processes connected to energy, glucose metabolism and immunity. In the case of obesity, adipose tissue produces proinflammatory cytokines such as TNF-α, IL-6 leptin, visfatin and angiotensin II, which modulate insulin resistance in two ways: directly, by signaling through the insulin pathway, and indirectly, by stimulating the inflammatory pathways (115). Obesity induces the accumulation of macrophages in adipose tissue, which influences inflammation and obesity-induced insulin resistance. In this context, the metabolically activated macrophage phenotype is produced by two independent mechanisms: one is mediated by palmitate internalization and the other involves palmitate binding to cell-surface TLRs. The balance between these two pathways determines the response of macrophages to metabolic dysfunction and can provoke differentiation to a proinflammatory or anti-inflammatory phenotype (116). As previously described, major ER functions include the production of lipids like cholesterol, glycerophospholipids, the synthesis and maturation of proteins and the regulation of calcium storage and dynamics. For this reason, recent studies have focused on the metabolic adaptation of adipose tissue macrophages (ATMs) in response to metabolic syndrome, and on how this affects the UPR pathway in macrophage polarization. It is generally accepted that obesity is associated with ER stress in adipose tissue and in local ATMs, which are key players in metabolic inflammation. ER stress boosts macrophage activation and the hyperactivation of the IRE1α-XBP1 axis in the adipose tissue of obese humans is known to disrupt the energy balance (117). Accordingly, recent studies have shown that blocking IRE1α alters the functional activities of macrophages, leading to changes in the M1-M2 polarization state. Work with IRE1α;Lyz2-Cre mice showed that specific IRE1α deletion in ATMs reduces the level of expression of proinflammatory marker genes in response to LPS, and increases polarization to the anti-inflammatory phenotype in response to IL-4. The M1-M2 imbalance of ATMs in turn severely limits the energy expenditure capacity of the mice by restricting the conversion of brown and beige fat, and also promotes insulin resistance (7). Thus, as abrogation of IRE1α in mice results in this broad range of metabolically beneficial effects, we suggest that it would be worthwhile exploring the role of the IRE-1α pharmacological inhibitor STF083010 by delivering it specifically to macrophages with the aim of reducing obesity.

To analyse the effect of the various branches of the UPR pathway, a recent study showed that macrophage-selective ablation of GRP78 in mice increased the level of expression of ATF4, promoting an M2 phenotype in macrophages, and boosted IL-6 production (118). A high-fat diet (HFD) up-regulates CHOP in adipocytes. Experiments in chimaeric mice revealed that M2 macrophages become significantly more numerous when recipients (adipocytes) are CHOP-deficient. These results suggest that CHOP in HFD induces a proinflammatory phenotype in ATMs and plays an important role in polarizing infiltrating macrophages in the case of obesity (119). Thus, the role of the UPR in other cell types may indirectly influence macrophage polarization.

On the other hand, ATF4 deficiency in mice is known to block insulin secretion and decrease insulin sensitivity in liver, fat and muscle. This function of ATF4 arises from its osteoblastic expression because osteoblast-specific ATF4 abrogation in mice shows the same metabolic abnormalities as occur in ATF4 KO mice. Additionally, ATF4 KO mice have a lower fat mass and lower blood glucose levels. At the molecular level, ATF4 induces the expression of the Esp gene in osteoblasts, which reduces the bioactivity of osteocalcin, an osteoblast-specific secreted molecule that enhances the secretion of, and sensitivity to, insulin (120). It is also known that HFD facilitates an increase in mitochondrial ROS levels and a metabolic adaptation that leads to TLR-induced UPR activation in dendritic cells. This activation increases IL-23 and IL-6 and modifies the innate immune response (121). Thus, the influence of UPR in these inflammatory contexts needs to be explored further to clarify its role in metabolism and macrophage polarization.

Finally, two studies have analyzed insulin resistance because it is associated with obesity and the role of UPR in the disease (122, 123). Obese mouse models revealed that ER stress contributes to insulin resistance and treatment with TUDCA, a chemical chaperone that enhances protein folding and alleviates ER stress, develops insulin sensitivity. Accordingly, a clinical trial has evaluated the effect of TUDCA in obese men and women, showing that treatment with TUDCA increases insulin sensitivity, but placebo treatment has not effect. TUDCA may therefore be an effective pharmacological therapy (124).

#### Fatty Liver

Non-alcoholic fatty liver disease (NAFLD) is considered to be a manifestation of liver metabolic damage as a result of insulin resistance and genetic susceptibility. Macrophages have been described as having an important function in the development and progression of this pathology as they are key cells in liver immune homeostasis (125). Indeed, an increase of ER stress was detected in Kupffer cells (KCs) in a mouse model of liver steatosis, which in turn helped aggravate liver damage upon ischemia-reperfusion injury (IRI). In this study, IRE1α knockdown in BMMs elicited an enhancement of anti-inflammatory “M2-like” macrophage polarization due to a decrease in STAT1 and STAT6 phosphorylation, while proinflammatory macrophage skewing was decreased. It is of note that adoptive transfer of IRE1α knockdown macrophages to mice with liver IRI under HFD can reduce organ damage (126). In addition, palmitic acid (PA) induces ER stress in macrophages, promoting a proinflammatory phenotypic shift in a PERK-dependent manner (127). In this study, PERK knockdown in macrophages inhibits the “M1-like” polarization induced by LPS/IFN and enhanced the M2-phenotypic shift induced by IL4. In contrast, other *in vitro* studies of RAW 264.7 macrophages demonstrated that PERK inhibition leads to a greater proinflammatory shift (37). Together, the results of these studies indicate that PERK has an important role in macrophage activation. However, there are some discrepancies regarding the type of polarization associated with PERK's functions, but these could have arisen because different chemical agents to induce ER stress and/or different macrophage stimulatory cues were used.

#### Atherosclerosis

In addition to obesity, macrophages are also critical determinants of the inflammatory process associated with atherosclerosis. The development of atherosclerosis is a complex process involving several metabolic and signaling pathways and is caused by the accumulation of cholesterol and oxidized low-density lipids (LDLs) in the arterial wall (128). As the disease progresses, monocytes adhere to the endothelium, invade it, and differentiate into macrophages, which ingest LDLs. These macrophages induce the formation of unstable plaques and a local proinflammatory microenvironment (129). In this context, various microenvironmental factors influence the macrophage metabolism and phenotype, which, in turn, produce changes in plaque evolution and progression. The atherosclerotic plaque is influenced by oxidative stress, hypoxia, hyperlipidaemia and cell death (130). In this pathology, the macrophage antioxidant response acts to reduce cellular ROS levels, which protects the mitochondria and other organelles from oxidative damage (131). In addition, the plaque milieu experiences local hypoxia due to an increase in the cellular metabolic demand, and the impaired oxygen delivery. This increases the level of HIF-1α, which is associated with angiogenesis (132). This atherosclerotic milieu is crucial to the metabolic reprogramming of macrophages and to their inflammatory response, which ultimately leads to disease progression and plaque instability. The vascular endothelium of these atherosclerotic lesions also has activated ER stress pathways, which are required for the production of proinflammatory cytokines that would further aggravate the damage generated by macrophages (133, 134). On the other hand, recent studies show that IRE1α is activated in lipid-laden macrophages that infiltrate this type of lesion. Treatment of BMMs with an IRE1α inhibitor suppresses activation of the NLRP3 inflammasome. This causes a significant drop in IL-1β secretion and could counteract the progression of atherosclerosis (8). Likewise, another study showed that ER stress is an essential regulatory pathway of macrophage plasticity and cholesterol metabolism. During macrophage differentiation from monocytes of obese individuals, pharmacological inhibition of ER stress with 4-phenylbutyrate (4-PBA) induces a shift to proinflammatory macrophages (135). This study used a non-specific ER stress inhibitor (4-PB4) and analyzed the PERK-ATF4-CHOP and IRE1α-XBP1 axes, but not that of ATF6. Therefore, more studies are needed using a specific inhibitor of ATF6 to analyse its involvement in macrophage phenotype shift in atherosclerosis. It is also known that the imbalance between lipid uptake and its efflux in macrophages is mediated by UPR in atherosclerosis, as specific CHOP knockdown in macrophages showed that lipid uptake promoted by ER stress is significantly reduced (136).

It is worth noting that several clinical trials are exploring the role of single UPR branches in diseases such as cancer, RA and type 2 diabetes (137). However, the rationale underlying these trials is not based on macrophage physiology. UPR can be targeted using either chemical inhibitors or small molecules inhibitors of the UPR signaling components (137). It is therefore crucial to address how to slow the progression of metabolic disorders by controlling ER stress-mediated macrophage plasticity. However, general conclusions cannot be drawn since the role of each UPR arm in macrophage polarization depends on its pathological context.

### Mechanisms of Hypoxia in Pathology and Macrophage Polarization

Hypoxia can be found in several specific pathological conditions such as tumors, atherosclerosis, rheumatoid arthritis (RA) and sites of bacterial infection. However, a hypoxic microenvironment characterizes virtually every site of inflamed, damaged or pathological tissue, due to the accumulation of large numbers of oxygen-consuming migrating inflammatory cells, local cell proliferation and vascular disruption (138) (Figure 2).

#### Cancer

The concentration of oxygen inside solid tumors is greatly reduced due to the rapid outgrowth and increased oxygen consumption rate of tumoral cells, which cannot be matched by blood supply. This intratumoral hypoxia is often associated with a malignant phenotype and poor prognosis in several types of cancer (139, 140), and hypoxia-driven accumulation of TAMs has been associated with a poor outcome (141–144). TAMs acquire pro-tumoral features and undergo metabolic adaptations fostered by the harsh tumor milieu, which include a combination of hypoxia, low nutrient levels, and accumulation of products derived from the altered tumor cell metabolism (145). Specifically, hypoxia induces the expression of migratory stimulating factors which act as potent chemoattractants to monocytes into the tumor site (146). Then, the hypoxic environment halts macrophage mobility and TAMs are trapped within the tumoral niche, where they are primed to serve protumoral functions and undergo metabolic reprogramming that shapes their functional phenotype including activation of glycolysis, alterations in their TCA cycle with subsequent use of alternative metabolites (such as glutamine), FA synthesis and altered nitrogen cycle metabolism (145). However, there is a broad heterogeneity of macrophage phenotypes within different tumors and tumor regions, and a distinction between M1- or M2-like macrophages cannot be inferred (5, 147, 148). Interestingly, hypoxia does not seem to influence differentiation of the MHCII-high (“M1-like”) or MHCII-low (“M2-like”) subsets, but does influence the MHCII-low macrophage phenotype by controlling hypoxia-responsive gene expression and promoting tumor progression through changes in metabolism, angiogenesis, and metastasis (149). Regarding this issue, several lines of work have investigated how TAMs could be molecularly repolarised to become proinflammatory, and thus antitumorigenic, through the action of a variety of molecules such as TLR agonists, cytokines (CSF2, IL12, etc.), antibodies, microRNAs, and so on. However, most of these molecules lack the potency required to act throughout the entire duration of the tumorigenic process, and many of them may provoke off-target inflammatory responses by generating systemic inflammation (150). It is therefore a matter of urgency to explore new ways of inducing macrophage repolarization toward an anti-tumoral phenotype.

#### Atherosclerosis

During the atherogenic process, as the plaque develops, thickening of the arterial wall results in reduced oxygen diffusion rates that, together with increased oxygen demand from infiltrating inflammatory cells, leads to the development of a hypoxic microenvironment (151). Thus, hypoxia has been proposed as an important contributor to the progression of human and murine atherosclerosis (152), and recent studies have drawn attention to the proatherogenic role of hypoxia in macrophage lipid and glucose metabolism (153, 154). In this context, foam cell formation is promoted by the increased expression of HIF-1α in intraplaque macrophages (155). Moreover, it has been demonstrated that the stabilization of HIF-1α and the increased expression of its downstream target genes take place within murine atherosclerotic plaques (154).

#### Rheumatoid Arthritis

On the other hand, RA is a chronic systemic autoimmune disease characterized by joint inflammation and destruction in which hypoxia is considered a relevant feature and a mechanism that determines disease pathogenesis (156). In fact, myeloid-specific deletion of HIF-1α in a murine model of arthritis markedly reduced joint swelling and synovial macrophage infiltration (157). Recent studies have detected that the imbalance between proinflammatory and anti-inflammatory macrophages, favored in a hypoxic environment, is one of the main causes of RA, where the proinflammatory cytokines secreted by the first aggravate RA symptoms, while anti-inflammatory cytokines released by the second alleviate them (158). Thus, drugs such as CP-25, which inhibits the production of TNF-α, IL-1β, and IL-6 produced by proinflammatory macrophages, have relieved the signs and symptoms of RA in animal models (159). Moreover, therapy with mesenchymal stem cells (MSCs) in the mouse model of collagen-induced arthritis (CIA) is very effective at lessening clinical symptoms by modulating macrophage polarization toward an anti-inflammatory phenotype (160). Lastly, the importance of hypoxia in the rheumatoid synovium has been highlighted by the proposed use of macrophages as cells that can deliver therapeutic genes under the control of a hypoxia-inducible promoter. Migration of these cells to synovial tissue would result in the transgene being switched on in diseased joints but not in healthy tissues (161).

#### Bacterial Infection

Finally, bacterial oxygen consumption and the formation of oxygen-impermeable biofilms help maintain a hypoxic environment during bacterial infection and promote the accumulation of phagocytes and vessel vasoconstriction in the area (162). HIF-1α has been associated with the bactericidal capacity of myeloid cells and is directly involved in the pathophysiology of sepsis (163, 164). Thus, HIF-1α-deficient cells are unable to mount proper anti-bacterial and inflammatory responses, due to the decreased expression of inducible nitric oxide synthase (iNOS) and the diminished production of glycolytic ATP (164). In this latter study, LPS-induced TNF-α secretion by macrophages was explored under normoxic and hypoxic conditions, which in both situations were equated after HIF-1α deletion. However, in WT cells, LPS-driven TNF-α secretion is augmented under hypoxia compared with normoxic conditions, meaning that HIF-1α adopts additional roles when oxygen is deprived upon LPS stimulation (157).

## Concluding Remarks and Perspectives

Although much work is still required to fully understand the metabolic molecular cues that drive macrophage polarization, compelling evidence summarized in this review suggests that the roles of hypoxia and the UPR are much more important than previously thought. An imbalance in the UPR components occurs in several pathological and inflammatory disorders, placing macrophages in maladaptive polarization states in which their metabolic balance is compromised. The contribution of hypoxia to the fine balance of the UPR regulatory arms is being explored, and it has been demonstrated that UPR activation influences hypoxia tolerance. It is known that the hypoxic pathological microenvironment can direct macrophage metabolism toward proinflammatory polarization that worsens the inflammatory response. Hence, as this hypoxic environment is closely linked to the UPR, which, in turn, is critical to the integration of the metabolic and inflammatory signals in macrophages, exploring this relationship may yield tools for designing more effective immunotherapies and for developing new strategies for reshaping macrophage phenotypes in several clinical settings. Many studies discussed in this review used very general UPR inhibitors, so before developing new clinical tools, it would be necessary to investigate animal models with specific deletions of the UPR individual components in macrophages, or develop macrophage-specific drug delivery systems, such as liposomes and nanoparticles. There are no clinical trials currently underway using specific inhibitors of the UPR pathways as treatment (just pan inhibitors such as the chemical chaperones TUDCA and PBA). In the case of HIF-1α, several clinical studies in cancer have been carried out, but the lack of selectivity of the inhibitors used increases the difficulty of correlating molecular and clinical responses in patients (165). Moreover, myeloid HIF-1α and HIF-2α may have non-redundant or even opposing functions in myeloid cell physiology (9). As such, discerning the roles of each isoform individually and developing selective isoform inhibitors should be a priority. Nevertheless, phenotypic conversion of macrophages offers attractive therapeutic targets and stress and oxygen-sensing pathways are clearly key determinants of their physiological and pathological functions. Moreover, outstanding questions regarding macrophage phenotypic conversion still need to be addressed and a close characterization of macrophage diversity within specific microenvironments must be further explored to elucidate the specificity of cellular metabolism of macrophages in each disease, for instance by using emerging sophisticated technologies such as single-cell analysis, multiplex immunohistochemistry and mass cytometry. Many questions arise concerning the clinical contexts in which UPR and/or HIF inhibitors are most likely to be therapeutically effective. Clinical translation will be challenging: how will it be possible to target these molecules in a myeloid-specific way, and how efficient will those therapies be? Future studies on these aspects will allow new treatment regimens to be developed for the broad spectrum of the aforementioned pathologies.

## Author Contributions

PD-B, MS, and RR designed and wrote the review. RR and CL-L coordinated and edited the manuscript. All authors contributed to the manuscript revision, and read and approved the submitted version.

### Conflict of Interest

The authors declare that the research was conducted in the absence of any commercial or financial relationships that could be construed as a potential conflict of interest.
